# Determination of the presence of 5-methylcytosine in *Paramecium tetraurelia*

**DOI:** 10.1371/journal.pone.0206667

**Published:** 2018-10-31

**Authors:** Aditi Singh, Adrienne Vancura, Rafal K. Woycicki, Daniel J. Hogan, Alan G. Hendrick, Mariusz Nowacki

**Affiliations:** 1 Institute of Cell Biology, University of Bern, Baltzerstrasse 4, Bern, Switzerland; 2 Graduate School for Cellular and Biomedical Sciences, University of Bern, Freiestrasse 1, Bern, Switzerland; 3 Tocagen Incorporated, San Diego, California, United States of America; 4 Storm Therapeutics Limited, Moneta Building, Babraham Research Campus, Cambridge, United Kingdom; Peking University Cancer Hospital and Institute, CHINA

## Abstract

5-methylcytosine DNA methylation regulates gene expression and developmental programming in a broad range of eukaryotes. However, its presence and potential roles in ciliates, complex single-celled eukaryotes with germline-somatic genome specialization via nuclear dimorphism, are largely uncharted. While canonical cytosine methyltransferases have not been discovered in published ciliate genomes, recent studies performed in the stichotrichous ciliate *Oxytricha trifallax* suggest *de novo* cytosine methylation during macronuclear development. In this study, we applied bisulfite genome sequencing, DNA mass spectrometry and antibody-based fluorescence detection to investigate the presence of DNA methylation in *Paramecium tetraurelia*. While the antibody-based methods suggest cytosine methylation, DNA mass spectrometry and bisulfite sequencing reveal that levels are actually below the limit of detection. Our results suggest that Paramecium does not utilize 5-methylcytosine DNA methylation as an integral part of its epigenetic arsenal.

## Introduction

The C5 position of cytosine can get methylated by DNA methyltransferases (DNMTs) into methylcytosine (5mC) and is necessary for the maintenance of chromatin structure, gene expression, and many chromatin-related processes[1–3]. 5mC has been extensively studied for its role in biological processes such as genomic imprinting, X chromosome inactivation and has long been implicated in human disease[3–8]. Furthermore, DNA methylation is also essential for sexual dimorphism[9], DNA repair[10] and for the suppression of transposable elements[11–13] in many organisms. DNA methylation is heritable and actively maintained by methyltransferases. However, it can be removed by the suppression of maintenance methyltransferases or by the action of specific demethylases. Cytosine methylation occurs mostly at the CpG or CNG sites in vertebrates and many other species across all phylogenetic kingdoms[14–17]. Cytosine methylation in motifs other than CpG and CNF has been shown in plants and *Drosophila* [18], [19]. Other eukaryotes such as *S*. *pombe*[20] and *C*. *elegans*[21] are devoid of cytosine methylation in their genome.

*Paramecium tetraurelia* is a unicellular eukaryote in the phylum ciliophoran exhibiting the characteristic nuclear dimorphism. *Paramecium* cells, like any other ciliate can go through either asexual or sexual way of reproduction. In *Paramecium*, one extensively studied phenomenon is its genome reorganization that occurs during the sexual life cycle[22]. During sexual cycle, the somatic macronucleus is degraded and lost, and a new somatic nucleus formed. During the new macronuclear development, somatic macronucleus is formed from the zygote that comes from cell’s germline-specific micronucleus. The formation of a functional somatic genome requires massive genome reorganization and the removal of germline-specific sequences[22] in macronuclear progenies of the zygotic nuclei.

The most remarkable example of such germline sequences is Internal Eliminated Sequences (IESs) that typically interrupt protein coding regions. Instructions for proper IES excision and ligation of gene segments is in part communicated from the maternal macronucleus during macronuclear development, though the specific mechanisms involved are still poorly understood[23].

Three alternative hypotheses attempt to explain the mechanism of IES targeting[24] through pathways involving a novel class of small RNAs, called scnRNAs. scnRNAs are generated from transcription of the germline genome during meiosis. The first hypothesis suggests scnRNAs direct histone modifications that mark IESs for elimination. Recent work suggests that histone modifications are scnRNA dependent and are required for IES excision[25]. However, most of the IESs in *Paramecium* are smaller than the size of a nucleosome[26], and hence this hypothesis seemingly cannot explain the precise targeting of smaller IESs. The second hypothesis suggests deposition of specific DNA modifications that mark IESs for excision (or gene segments for retention). The macronuclear genome does contain N6-methyladenosines, but the presence of 5mC is still not clear[27]. The third hypothesis suggests that the scnRNAs themselves directly help in the targeting of IESs for excision. The primary challenge to this hypothesis is the presence of IESs whose precise excision is scnRNA independent.

Indirect evidence using cytosine analogs suggests that cytosine methylation might be present in the genome[28], [29] even though homologs of canonical DNA methyltransferase are seemingly absent. These studies argue that the somatic nucleus is programmed by 5-methyl cytosines that leads to the repression of certain somatogenic sequences during sexual cycle. The argument is based on the findings where administration of 5-azacytidine during sexual reproduction in *Paramecium* alter expression of certain somatogenic sequences in the subsequent asexual cycles. Furthermore, recent study in another ciliate *Oxytricha trifallax* also showed evidence for the presence of methylated cytosines in the genome using mass-spectrometry and bisulfite sequencing[30]. In order to clarify this paradox and refine potential models for DNA elimination we measured the levels and locations of DNA 5mC in *Paramecium tetraurelia* using multiple methods.

## Materials and methods

### Culture conditions for *Paramecium tetraurelia*

*Paramecium tetraurelia* strain 51 cells, mating type 7, were used for the experiments. *Paramecium* cells were grown in 1x wheat grass powder (WGP, Pines International) bacterized with a non-virulent strain of *Klebsiella pneumonia*. β-Sitosterol, essential for *Paramecium* growth (0.8 mg/l), was added to WGP medium just before the feeding the cells[31]. For DNA mass spectrometry, cells were fed with DCM^-^ *E*. *coli* (C29251, NEB) instead of *Klebsiella pneumonia*. Cells were either cultured at 27°C or 18°C as per requirement.

### Colorimetric quantification of methylated DNA

Total genomic DNA was extracted from different developmental stages after the treatment with RNase A (R6148, Sigma) treatment using phenol: chloroform: IAA. The colorimetric quantification of 5-methyl cytosines in total genomic DNA was done using EpiSeeker methylated DNA Quantification Kit (ab117128, Abcam). Briefly, DNA is bound to strip wells (provided with the kit) having a high DNA and the methylated DNA is captured and detected using antibodies that are then quantified by reading the absorbance in a microplate spectrophotometer. The amount of methylated DNA is proportional to the measured OD intensity.

### Immunofluorescence

100 μl of cells from each time point were transferred to a 1.5 ml tube and washed twice with 1ml of 1x PBS after the incubation for 5 minutes each. The cells were then centrifuged at 350rcf for 2 min. After removing the supernatant, cells were treated with 1 ml Triton X-100 in PHEM (5x PHEM: 50 mM EGTA, 125 mM HEPES, 10 mM MgCl2, 300 mM PIPES) and incubated for 20 min for permeabilization. Cells were then treated with,2% PFA in PHEM for 15 min, followed by two PBS washes. After that, the cells were incubated in 1 ml of 0.1 M HCl for 5 min and then washed again with 1XPBS. Cells were then blocked for 20 min with 1 ml of 3% BSA (3% BSA+ 0.1% Triton X-100 in TBSTEM (10 mM EGTA, 2 mM MgCl2, 0.15 M NaCl, 10 mM Tris, pH adjusted to 7.4. Tween 20 (1%, v/v)). For primary antibody anti-5-methylcytosine (Abcam, ab73938) in the mouse was used in a 1:100 at room temperature. The cells were washed twice in PBS for 10 min and then the secondary antibody, goat-anti-mouse Alexa Fluor 488 (Invitrogen) was added to a final volume of 500 μl. Cell samples were incubated for 1.5 h at 37°C. The cells were rewashed twice in PBS for 10 min and then DAPI (1 μg) stained in 1 ml BSA Mix for 3 min. 20μl mounting medium (Prolong Gold Antifade, Invitrogen) was added into the samples and the slides were sealed using cover slips.

### Confocal microscopy

Confocal images were taken on an Olympus Fluoview FV1000 confocal microscope. For the detection of 5mC, images were taken with UPLSAPO40X2 with numerical aperture 0.95, whereas, images in Azacytidine inhibitor experiment were taken with UPLSAPO60XO with numerical aperture 1.35. Images were analysed using the software Imaris (Bitplane) and ImageJ[32].

### Inhibition by Azacytidine and Decitabine

For the inhibition assay 5-Aza-2’-cytidine (Sigma) was used in two different concentrations of 10μM and 3 μM whereas 5-Aza-2’deoxycytidine/Decitabine (Sigma) was used in three different concentrations of 1.5μM, 1 μM and 0.5 μM. Both Azacytidine and Decitabine were added to the 0.2x WGP bacterized media. The culture media was changed daily, and the cells were treated for four consecutive days until the cells reached 20 vegetative cycles, making them be able to go through autogamy.

### Survival test and IES retention PCR

After cells had finished autogamy, 30 post-autogamous cells were transferred into medium bacterized with K.*pneumoniae* for the survival test. Cells were monitored for three days (approximately 12 divisions) and categorized into three groups according to the observed phenotype. In parallel, 100ml culture was harvested for DNA extraction using GeneElute–Mammalian Genomic DNA Miniprep Kit (Sigma-Aldrich). PCRs were done on different genomic regions flanking an IES with GoTaq polymerase (Promega). Agarose gel images were taken on Alpha Innotech MultiImage II AlphaImager HP.

### Genomic DNA extraction

Cells were harvested and washed twice with 10mM Tris-HCl pH 7.4. The cell pellet was then incubated with the lysis buffer. Cells were incubated with 20μl of RNase A (from GeneElute–Mammalian Genomic DNA Miniprep Kit Sigma-Aldrich) for five minutes at room temperature. Afterwards, cells were incubated in three volumes of the lysis buffer (0.5M EDTA, 1% SDS, 1% N-lauryl sarcosine, 1 mg/ml of proteinase K) at 55°C for overnight. The cells were then incubated for 1Hr with an equal volume of Phenol: Chloroform: IAA on a shaker with gentle shaking. After incubation, the cells were centrifuged at the maximum speed of the table top centrifuge for 10minutes. The supernatant was then transferred to a clean tube, and genomic DNA was precipitated using 800μl of isopropanol.

The T47D human cell line was purchased from Sigma (catalogue number: 85102201) and the MCF7 human cell lines were purchased from ATCC (catalogue number: HTB-22D). DNA was prepared using a Zymo Research Kit (catalogue number: D4068).

### Global quantification of 5-methylcytosine using DNA mass spectrometry

Measurements of 5-methylcytosine levels were performed by Zymo Research (http://www.zymoresearch.com) using mass spectrometry. An SRM-based mass spectrometry assay was used to quantify 5-hydroxymethyl-2’-deoxycytidine (5HmdC) and 5-methyl-2’-deoxycytidine (5mdC). The assay was designed to measure 5HmdC concentrations and 5mdC concentrations as a percentage of 2’-deoxyguanosine (dG) (e.g.–[5HmdC]/[dG] and [5mdC]/[dG]). The calibrated ranges for the analytes were 0–2.5% for 5HmdC and 0–25% for 5mdC using a fixed 40 pmol amount of dG as an internal standard.

Positive controls together with Dev1 genomic DNA samples were sent to Storm Therapeutics Limited where they were enzymatically digested to individual nucleosides according to a previously optimised protocol[33]. The samples were analysed in both full-scan- and PRM-mode in an Orbitrap QExactive-HF High-Resolution Mass Spectrometer (Thermo Fisher, Waltham, Massachusetts, USA). Standard curves were also run indicating a lower limit of detection of 300pg/ml for each nucleoside.

### Bisulfite sequencing analysis

For bisulfite sequencing, total genomic DNA extracted from different time points were sent to the Lausanne Genomic Technologies Facility at the University of Lausanne. Library preparation with bisulfite conversion was performed with the Ovation Ultralow Methyl-Seq kit (Nugen) after spiking in Phi-X lambda DNA to ~0.5% total mass. Paired-end sequencing was performed on Illumina HiSeq 2500. Sequencing reads in the form of fastq files were processed following the suggested protocol on Bismark website using version 0.18.1 (https://rawgit.com/FelixKrueger/Bismark/master/Docs/Bismark_User_Guide.html). First, reads were quality trimmed and filtered using Trim Galore[34], [35]. Filtered reads were then mapped to the *Paramecium* macronuclear genome (ptetraurelia_mac_51.fa) with options–n 1 and–X 1000. Methylation calls were then extracted for all C’s with bismark_methylation_extractor command. Bismark Cytosine reports were combined, processed and analyzed in R.

Human methylation dataset (SRR34552)[36] was used as a positive control for the analysis workflow. Reads were mapped to human GRC37.

### Bisulfite PCR

We used the EpiTect Fast Bisulfite Kit (59802, Qiagen) to do bisulfite conversion on total gDNA. Primer pair optimized for Bisulfite converted DNA was designed flanking the loci predicted to have 5-methylcytosine. Primer pair 5’-ATG GTA TTT AGA TAA TTA TAT GGG-3’/ 5’-TAT AAA CGA ACA AAT TAA ATA AAC C-3’ was used for PCR amplification. PCR product was then purified using Wizard SV Gel and PCR Clean-Up System (Promega) and samples were sent for Sanger sequencing at Microsynth.

## Results

### Colorimetric and immunofluorescence assays suggest the presence of cytosine methylation during autogamy

**Fig 1:**
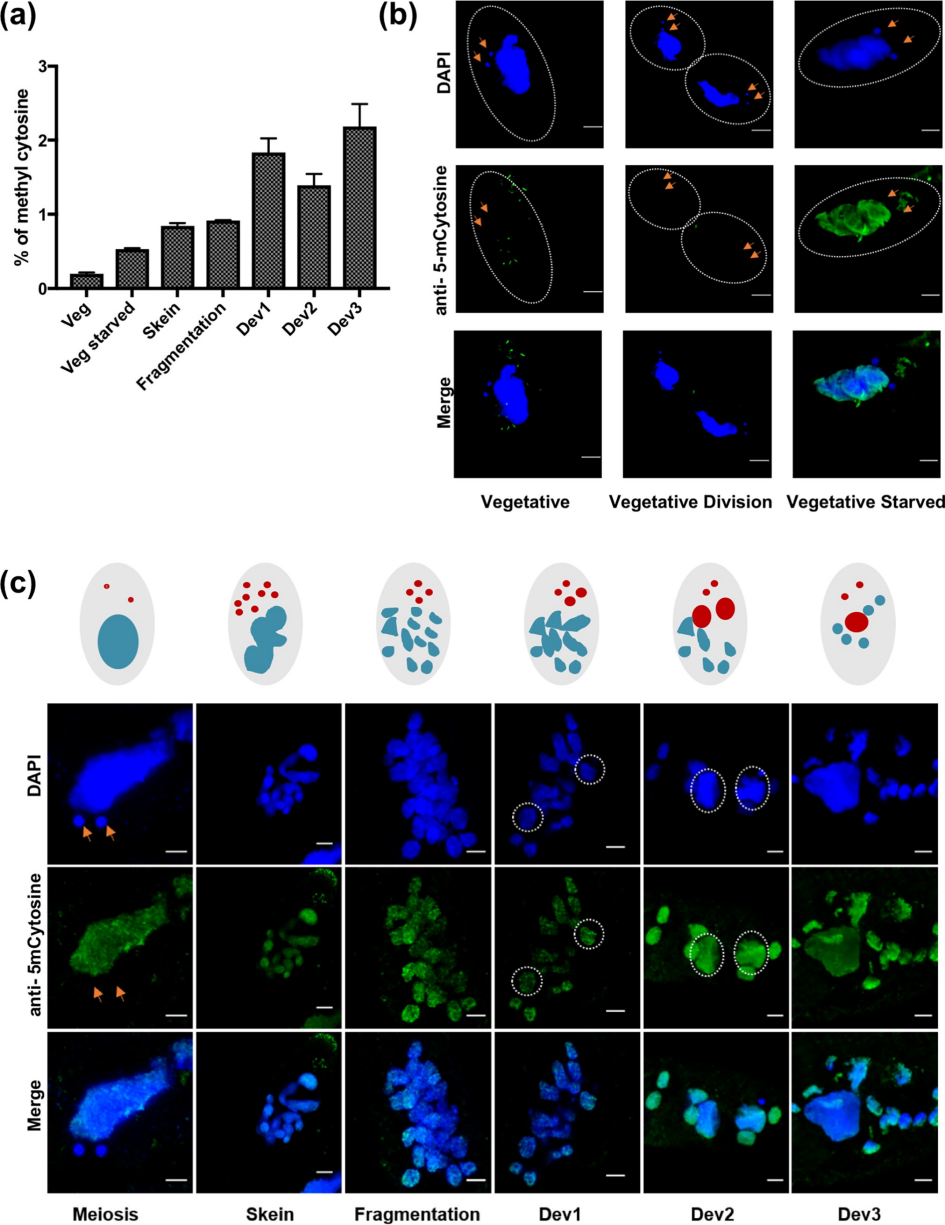
Detection of 5-methylcytosine by the antibody-based method. (a) Colorimetric assay for total 5-methylCytosine; total gDNA of different developmental stages was extracted and the assay was carried in biological triplicates, error bar represents the standard deviation, (b) Immunocytochemistry with antibody against 5-methylcytosine during vegetative growth; veg represents wild type vegetative cells, vegetative Division represents a cell going through cytokinesis during vegetative division, Vegetative starved is the stage where wild type cells were allowed to make four divisions and then was starved for 24hrs to block further vegetative divisions, (c) Schematic representation of autogamy in *Paramecium*; red represents micronuclei and the developing new macronucleus, blue represents macronucleus, (d) Immunocytochemistry with antibody against 5-methylcytosine during vegetative growth; Meiosis represents beginning of micronuclear meiosis, Skein represents beginning of the fragmentation of parental macronucleus, Fragmentation represents a population where about 40% of cells have fragmented parental macronucleus, Dev1 is the stage during autogamy when most of the cells have visible new macronucleus, Dev 2 is the stage during autogamy when majority of cells have fully developed new macronucleus ready for karyonidal division, dev 3 is after post karyonidal divison. Orange arrows represent micronucleus, dotted circles show new macronucleus. Scale bar: 5μm.

There are no known cytosine methyltransferases encoded in the *Paramecium* genome to investigate for a functional role during autogamy. This is also the case for other ciliates whose genomes have been sequenced, including *Oxytricha trifallax*, where cytosine methylation was recently reported[30]. To detect the presence of 5mC in *Paramecium*, we used the EpiSeeker methylated DNA Quantification Kit (Abcam). Genomic DNA was extracted from vegetative cells, from vegetative but starved cells and from cells at various developmental stages. Cytosine methylation was detected as soon as the cells sensed starvation (either vegetative or those induced to go through autogamy) and continued to be present at low levels during the early stages of autogamy. The levels were highest in the later stages when the new macronuclei are visible in the cells (Fig 1A).

Immunostaining was performed with an antibody against 5mC on cells harvested and fixed during vegetative growth and during autogamy. 5-methylcytosine immunofluorescence was not observed during any stages of the vegetative life cycle; neither in the macronucleus nor in the micronucleus (Fig 1B). However, as soon as the cells were starved, 5mC signal appeared in the macronucleus. The signal appears to be dependent on starvation since it is also present in young cells that are not mature enough to go through autogamy. During autogamy, we observed 5mC antibody signal in the parental macronucleus as early as the beginning of meiosis, and the signal remained present as long as the parental macronuclear fragments were present in the DAPI-stained cells (Fig 1C). Moreover, antibody signal was also observed in the developing macronucleus as soon as the Dev1 stage and was present until Dev3 stages (Fig 1C). As a control, we performed immunostaining with only secondary antibody and we did not detect any signal in the nucleus (S1A Fig). We also used *C*.*elegans* embryo that do not contain 5mC and human embryonic kidney cells (HEK 2939) reported to have the methylation as controls for our immunofluorescence studies (S1B & S1C Fig, respectively). These results provide indirect evidence for 5mC incorporation during macronuclear development.

### Treatment with Azacytidine and Decitabine affects cell growth

Given the positive 5mC signals during autogamy, we wanted to see if its deposition is necessary for DNA elimination. Azacytidine and Decitabine (deoxycytidine) are well known nucleosidic and deoxynucleosidic analogs of cytidine, respectively. Neither of these drugs can be methylated as they contain a nitrogen at position 5 of the pyrimidine ring. They can also be easily incorporated into DNA during replication. Additionally, these drugs form a covalent bond with DNA methyltransferase leading to the degradation of these enzymes, and thus can cause general inhibition of methylation processes[37], [38]. To look for a possible role of cytosine methylation, we did a chemical treatment of cells with both Azacytidine and Decitabine. The effective concentration of both the drugs is known[39–41], 3μM for Azacytidine and 1μM for Decitabine. A *Paramecium* cell undergoes four vegetative divisions per day under normal conditions. First, we tested if drug treatment had any afffect on vegetative cells. Cells were treated with either Azacytidine (3μM or 10μM) or Decitabine (0.4μM, 1μM or 1.5μM) for five consecutive days. We did not observe any morphological or growth defect during the treatment, and the cells underwent their normal rate of four divisions per day.

Drug treatment lead to a decrease in 5mC detection via immunofluorescence (S2A Fig, middle panel). However, we did not observe significant reduction in 5mC signal after decitabine treatment using immunofluorescence (S2A Fig, right panel). After the treatment, the cells were induced to go through autogamy. Furthermore, 30 individual cells were isolated to test if they could go through the vegetative cell cycle after autogamy. With 3 μM Azacytidine treatment, about 25% cells could not survive after autogamy, whereas, about 35% of cells did not make the typical four divisions per day (S2B Fig). In the case of Decitabine treatment, only a mild growth defect was observed, and none of the treatments were lethal (S2D Fig).

To test whether the treatment of Azacytidine or Decitabine affects DNA elimination during sexual cycle, we used primers flanking the IES regions and performed PCR to check for IES retention. We could not observe any IES retention after the treatment either of the drugs, suggesting that the treatment with a nucleosidic analog does not affect DNA elimination in the developing macronucleus (S2C & S2E Fig full length gels in supp. S3 and S4 Figs).

### DNA mass-spectrometry analysis to detect DNA 5-methyl cytosine methylation

Since immunostaining and a commercial kit suggested the presence of methyl cytosines, we wanted to assess the total percentage of methyl cytosine in the genome. For this, we sent total genomic DNA isolated from cells starved during vegetative divisions (vegetative starved) and cells regularly fed vegetatively dividing cell cultures (vegetative unstarved), from a cell culture containing cells where 40% of the population had fragmented parental macronucleus during autogamy for DNA mass spectrometry (Zymo Research). We also sent total genomic DNA isolated from a cell culture where most of the cells had visible new macronucleus during autogamy and a post-autogamous culture for DNA mass spectrometry.

**Fig 2:**
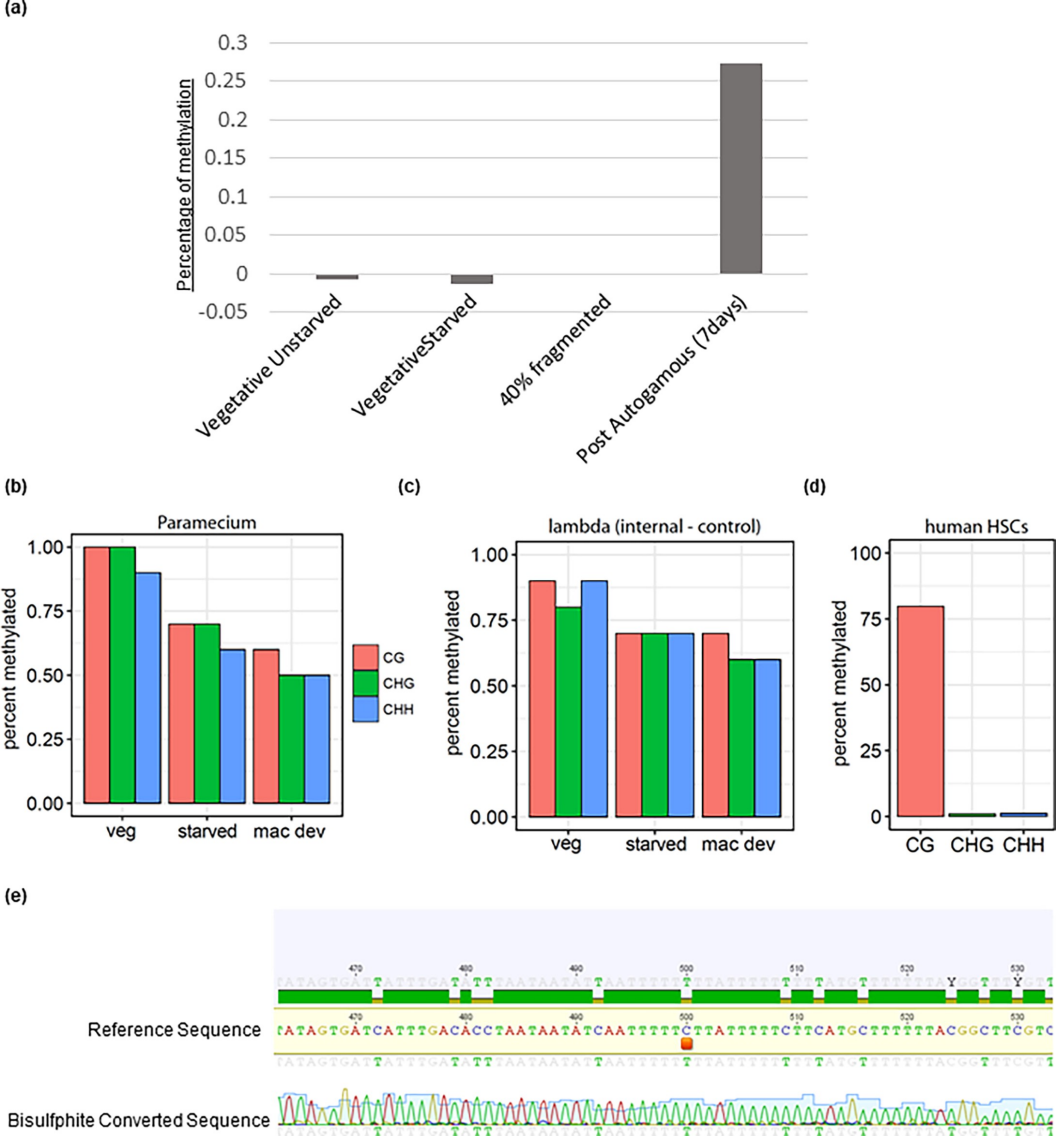
5-methylcytosine detection using mass spectrometry and bisulfite sequencing. (a) DNA mass spectrometry analysis showing the percentage of cytosine methylation, vegetative unstarved; wild type cells were allowed to undergo vegetative divisions, starved vegetative; wild type vegetative cells grown four divisions and then starved for 24hours before extracting gDNA, 40% fragmented; a population where about 40% of cells have fragmented parental macronucleus during autogamy, post-autogamous; cells after seven days post autogamy. (b) Bisulfite sequencing analysis on different developmental stages; veg represents wild type vegetative cells, Veg starved is the stage where wild type cells were allowed to make four divisions and then was starved for 24hrs to block further vegetative divisions mac dev represents 40% fragmented, (c) Lambda DNA was spiked in sequencing samples as a negative control. (d) raw data from a published human bisulfite dataset was downloaded and ran through the workflow. (e) snapshot of mapping of putative loci for methylated cytosine (marked with orange bar) with the reference genome. Genomic DNA was treated with Epitect Fast DNA Bisulfite Kit (Qiagen) for Bisulfite conversion, PCR product was then sent for Sanger sequencing and mapped with Geneious software version R8.

An SRM-based mass spectrometry assay was used to quantify 5-hydroxymethyl-2’-deoxycytidine (5HmdC) and 5-methyl-2’-deoxycytidine (5mdC). The assay was designed to measure 5HmdC concentrations and 5mdC concentrations as a percentage of 2’-deoxyguanosine (dG) (e.g.–[5HmdC]/[dG] and [5mdC]/[dG]). Methyl cytosines were only detected in the postautogamous culture and to only about 0.3% genome-wide. Thus, the mass spectrometry results do not corroborate the immunostaining or colorimetric measurements (Fig 2A). As controls, we also performed DNA mass spectrometry on genomic DNA isolated from *Drosophila*, *E*. *coli*, human breast adenocarcinoma MCF-7 and T47F cell lines. Methyl cytosine levels from controls matched previously reported values[42–44] (S5 Fig).

### Bisulfite sequencing analysis suggests DNA cytosine methylation is rare or non-existent

We performed bisulfite converted paired-end sequencing on the samples described above and spiked-in a small amount of lambda DNA as an internal negative control. Sequencing reads were processed using the Bismark workflow[45] and mapped to the *Paramecium* macronuclear genome and lambda genome. As a positive control of the sequencing workflow, we downloaded the raw data from a published human bisulfite dataset[36] and analyzed it in parallel.

The paucity of filtered and quality trimmed reads, as well as the high mapping rate to the macronuclear genome (>60%), attest to the quality of the sequencing dataset. Bisulfite sequencing relies on the conversion of cytosines to uracil residues but 5-methyl cytosines do not get converted to uracils. Across all mapped sequenced bases, < 1% of C’s were not converted to T’s (and thus potentially methylated) in all conditions for each of the three sequence contexts (CG, CHH, CHG–Fig 2B). However, nearly identical results were obtained from the internal negative control lambda genome (Fig 2C). In contrast, ~75% of Cs in CpG context were called as C from the control human HSC dataset, consistent with the published findings[36] (Fig 2D). These results validate the sequencing workflow and suggest that if there is cytosine methylation in *Paramecium* macronuclear genome, it is a rare event.

For each cytosine in the *Paramecium* macronuclear genome, we plotted the percent not converted to thymine. For these analyses, we removed sites in the genome with less than 10X coverage in all three conditions. The results for each of the three conditions are nearly identical (S6 Fig, left panel): ~80% of sites had zero not converted C’s, ~19% had less than 10% not converted C’s and less than 1% had greater than 10% not converted C’s. There were 340 sites (0.002%) with greater than 30% converted C’s in vegetatively grown cells and none in either starved cells or cells undergoing macronuclear development. There was no bias in C conversion among the three different nucleotide contexts (S6 Fig, right panel)

Furthermore, we tested specific loci using the Epitect Fast DNA Bisulfite Kit (Qiagen) followed by Sanger sequencing. For all tested loci, cytosines were converted to thymines, suggesting that they are not methylated (Fig 2E). Therefore, we conclude that if there is DNA 5-methylcytosine in the *Paramecium* macronuclear genome its abundance is below the limit of detection of the current techniques available, and unlikely to have a major role.

## Discussion

DNA methylation is important for differentiation and development in plants and animals[1], [46], [47]. Though there was no clear evidence of presence or absence of cytosine methylation in *Paramecium*, recent studies identified its presence in *Stylonychia lemnae*[48] and *O*. *trifallax*[30]. In both the cases, cytosine methylation was shown to take an active part in macronuclear development, albeit, using different modes. In *Stylonychia*, it was suggested that demethylation of two specific promoters correlates with the activation of genes required for macronuclear differentiation[49]. On the other hand, *Oxytricha* uses *de novo* cytosine methylation for DNA elimination during conjugation[30]. Apart from these examples, two more ciliates have been reported to possess cytosine methylation in their genomes[50], [51]. However, in none of the cases, has the cognate DNA methyltransferase been identified. The current knowledge of the presence of methylated cytosines and their potential role in macronuclear development encouraged us to investigate the possible presence of cytosine methylation in *P*. *tetraurelia* as well.

Although our preliminary data using the antibody-based methods (Fig 1) seemed encouraging and suggested the presence of 5mC in *Paramecium*, these results were contradicted by DNA mass spectrometry and bisulfite sequencing analyses (Fig 2A and 2B–2D). It’s thus likely that the antibody-based methods have some spurious background signal, such as detection of methylated RNA or of other unidentified molecules unrelated to 5mC. We were not able to detect any signals using a different anti-5mC antibody (S1D Fig). Therefore, quantification of 5mC by immunofluorescence and methylated DNA immunopurification (MeDIP), which rely on the use of antibodies, may give false positives, especially if actual 5mC levels are low. Together with other techniques, the detection of cytosine methylation in *Oxytricha* [30] is also based on these two methods. Therefore, it is essential to take a critical look at the data to infer the rate of false positives to attain the accurate picture of methylation levels within the genome. On the other hand, even though bisulfite conversion and sequencing are the gold standard in detecting 5mC, this method can also give false positives through incomplete conversion. Thus, including an internal negative standard, such as lambda DNA, is essential to identify the sensitivity of each experiment.

DNA mass spectrometry is considered to be a highly sensitive method to detect DNA methylation, and indeed showed the presence of cytosine methylation at very low levels in the postautogamous culture (Fig 2A). Here, we cannot rule out the possibility of bacterial contamination even though the cells were fed with DCM^-^ *E*. *coli* strain that does not undergo DNA methylation. The rate of bacterial contamination in the postautogamous cultures may be higher owing to a longer incubation period. DNA mass spectrometry or high-performance chromatography methods are based on the detection of methylation on digested mononucleotides. Thus, we cannot exclude the possibility that the signal detected by these analyses might come from the residual food in the culture, especially when the detected levels are quite low[48], [50].

In light of our 5mC results, the effect on cell growth due to the treatment with Azacytidine or Decitabine (S2 Fig) cannot be attributed to hypomethylation or demethylation, at least completely. It is known that these analogues can alter gene expression indirectly and not necessarily always by promoter hypomethylation[52]. Furthermore, the drugs could have other cytotoxic effects in *Paramecium*. The IESs tested with PCR did not show detectable 5mC, suggesting that macronuclear development may not be affected *per se*.

In summary, our current study suggests that if 5mC is present in the *Paramecium* genome its levels are below the current limits of detection (< 1%). Moreover, even if 5mC is present at low levels, its unlikely to have a role directing IES excision.

## Supporting information

S1 Fig Effect of inhibitor treatment on cell survival and IES retention. Immunofluorescence using; (a) only Alexa Fluor 488 secondary antibody on a population of *Paramecium* where majority of cells have fragmented parental macronucleus,(b, left panel) *C*.*elegans* embryo stained against 5- mCytosine as a negative control for immunofluorescence, (c, right panel) Human embryonic Kidney cells stained against 5-mCytosine (Abcam, ab73938) as a positive control for immunofluorescence, (d) immunofluorescence against 5-mCytosine (Diagenode, C15200081) during early and late stages of macronuclear development. Scale bar: 5μm for a, b and c, 7μm for d.
(PDF)

S2 Fig Effect of inhibitor treatment on cell survival and IES retention. a) Immunocytochemistry with antibody against 5-methylcytosine after Azacytidine and Decitabine treatment for three consecutive days. Scale bar: 5μm. (b) & (d) Survival test on cells treated with Azacytidine/ Decitabine; sick; cells did not undergo normal vegetative division rate, dead; non-viable progenies after refeeding, normal; sexual progenies that underwent normal division rate after refeeding. (c) & (e) IES retention PCRs on different loci (full-length gels are presented in S3 & S4 Figs respectively) with the primers flanking an IES region (S1 Table).
(PDF)

S3 Fig Full length gels on IES retention PCRs corresponding to S2C Fig. (PDF)

S4 Fig Full length gels on IES retention PCRs corresponding to S2E Fig. (PDF)

S5 Fig Percentage of methylation calculated after mass spectrometry done on total genomic DNA samples from different Paramecium during autogamy when new macronuclei are observed in the cell. Drosophila, E. coli, Human MCF7 DNA and Human T47D DNA (provided by Storm Therapeutics Limited) were used as a positive control for the detection of methylated cytosines.
(PDF)

S6 Fig Absence of evidence of C methylation in *Paramecium* mac genome. Left panel, percent calculated using C/(C + T) for each C in *Paramecium* genome. There was no bias in C conversion among the three different nucleotide contexts (right panel).
(PDF)

S1 Table List of primers. List of primers to check IES retention PCRs.
(PDF)
