# Correction: Determination of the presence of 5-methylcytosine in *Paramecium tetraurelia*

**DOI:** 10.1371/journal.pone.0209707

**Published:** 2018-12-20

**Authors:** Aditi Singh, Adrienne Vancura, Rafal K. Woycicki, Daniel J. Hogan, Alan G. Hendrick, Mariusz Nowacki

In the Author Contributions section, Rafal K. Woycicki (RKW) should be listed as one of the persons that completed Formal Analysis for the paper.
