# Supplementary material for: Determination of the presence of 5-methylcytosine in Paramecium tetraurelia
Source: PLoS One. 2018 Oct 31;13(10):e0206667. doi: 10.1371/journal.pone.0206667 (PMC6209305; doi:10.1371/journal.pone.0206667)
Supplement: S1 Fig — Immunofluorescence using; (a) only Alexa Fluor 488 secondary antibody on a population of Paramecium where majority of cells have fragmented parental macronucleus,(b, left panel) C.elegans embryo stained against 5- mCytosine as a negative control for immunofluorescence, (c, right panel) Human embryonic Kidney cells stained against 5-mCytosine (Abcam, ab73938) as a positive control for immunofluorescence, (d) immunofluorescence against 5-mCytosine (Diagenode, C15200081) during early and late stages of macronuclear development. Scale bar: 5μm for a, b and c, 7μm for d. (PDF) [file pone.0206667.s001.pdf]

S1 Fig

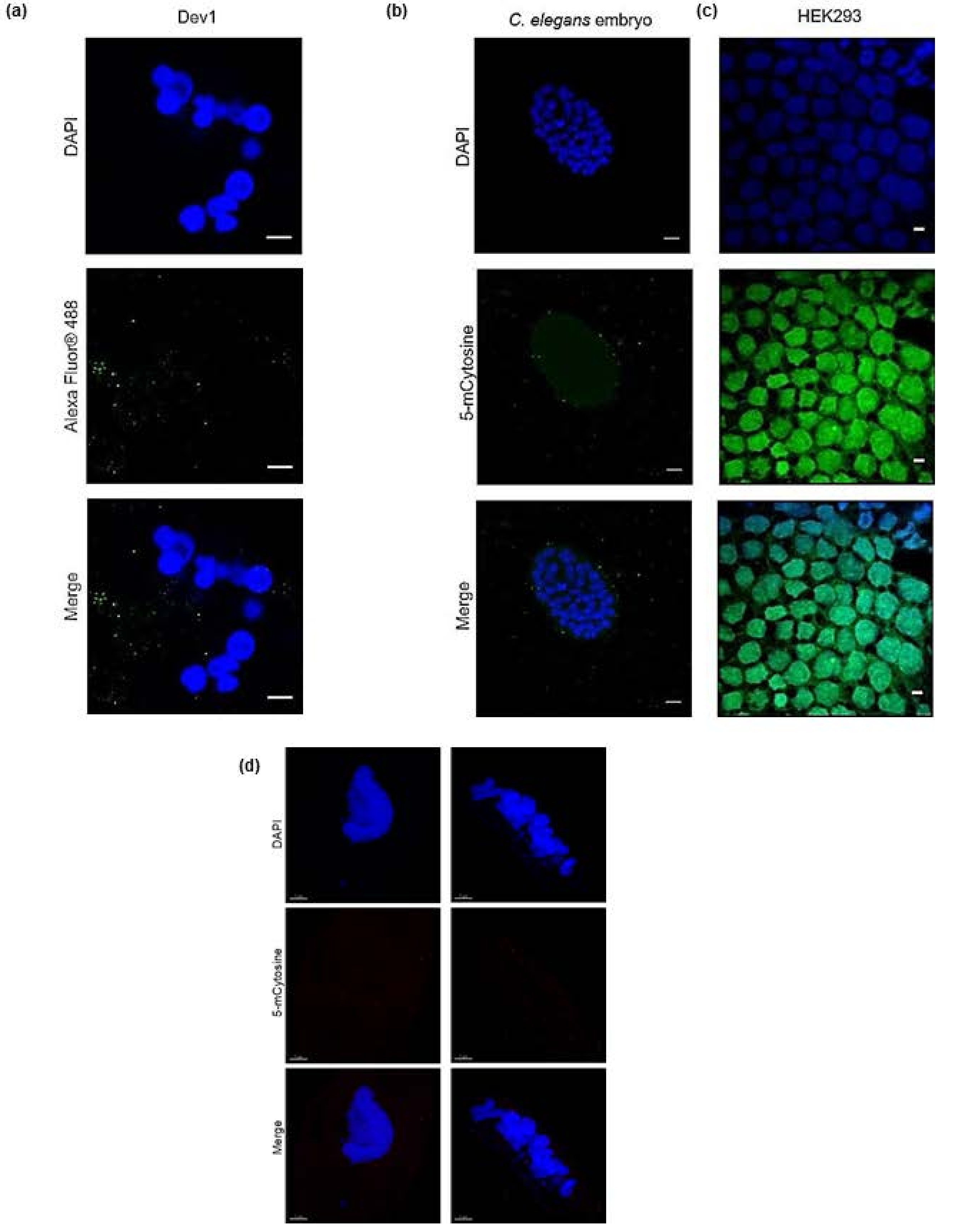

S1 Fig. Immunofluorescence using; (a) only Alexa Fluor 488 secondary antibody on a population of *Paramecium* where majority of cells have fragmented parental macronucleus,(b, left panel) *C.elegans* embryo stained against 5- mCytosine as a negative control for immunofluorescence, (c, right panel) Human embryonic Kidney cells stained against 5-mCytosine (Abcam, ab73938) as a positive control for immunofluorescence, (d) immunofluorescence against 5-mCytosine (Diagenode, C15200081) during early and late stages of macronuclear development. Scale bar: 5µm for a, b and c, 7µm for d.
