# Supplementary material for: Determination of the presence of 5-methylcytosine in Paramecium tetraurelia
Source: PLoS One. 2018 Oct 31;13(10):e0206667. doi: 10.1371/journal.pone.0206667 (PMC6209305; doi:10.1371/journal.pone.0206667)
Supplement: S2 Fig — a) Immunocytochemistry with antibody against 5-methylcytosine after Azacytidine and Decitabine treatment for three consecutive days. Scale bar: 5μm. (b) & (d) Survival test on cells treated with Azacytidine/ Decitabine; sick; cells did not undergo normal vegetative division rate, dead; non-viable progenies after refeeding, normal; sexual progenies that underwent normal division rate after refeeding. (c) & (e) IES retention PCRs on different loci (full-length gels are presented in S3 & S4 Figs respectively) with the primers flanking an IES region (S1 Table). (PDF) [file pone.0206667.s002.pdf]

S2 Fig

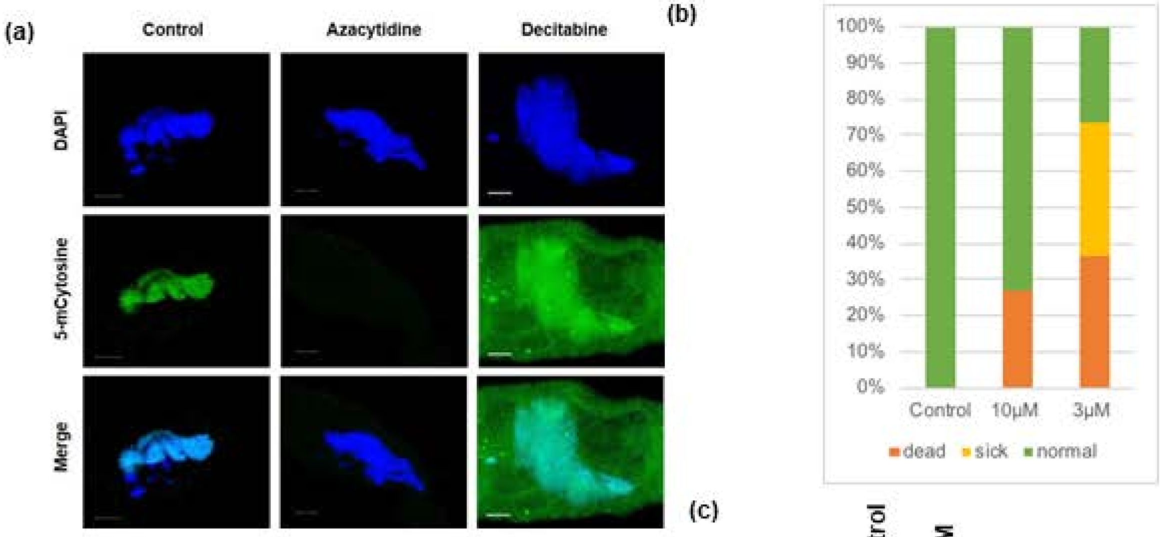

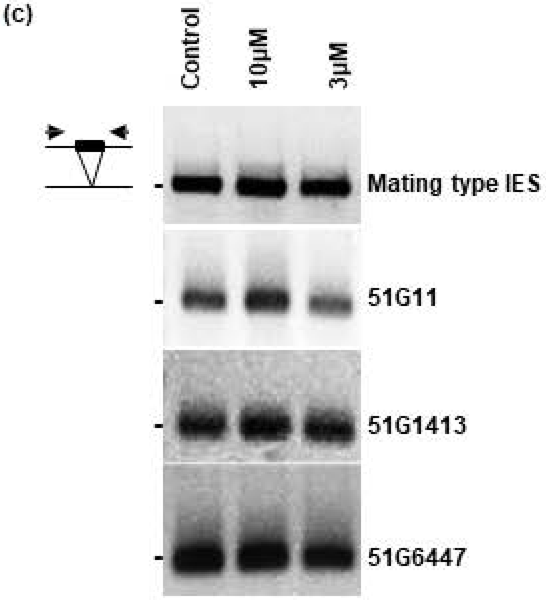

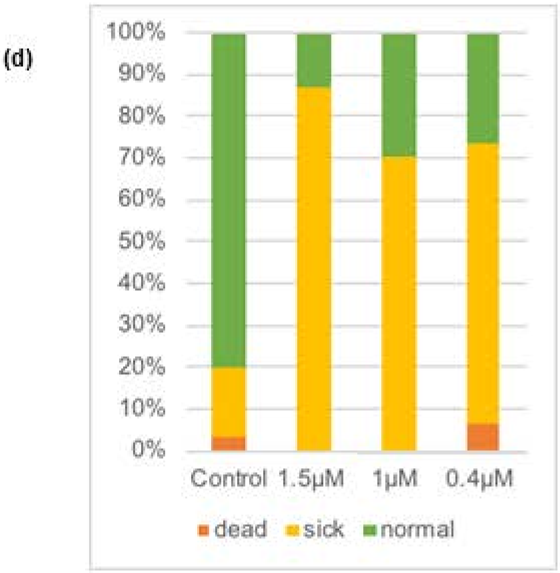

S2 Fig. Effect of inhibitor treatment on cell survival and IES retention.

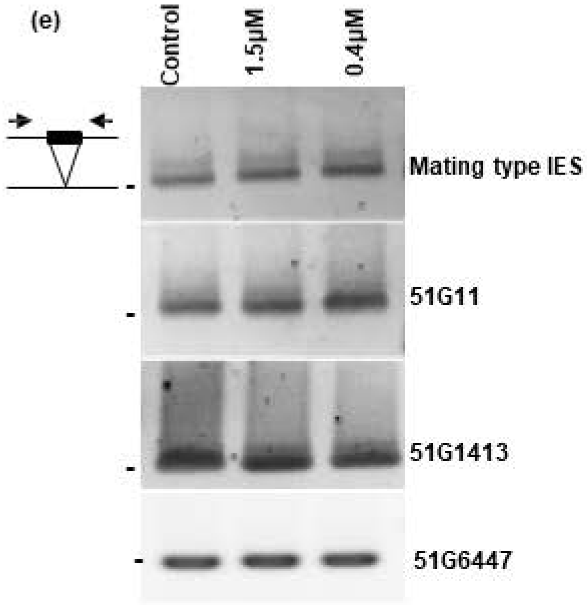

(a) Immunocytochemistry with antibody against 5-methylcytosine after Azacytidine and Decitabine treatment for three consecutive days. Scale bar: 5µm. (b) & (d) Survival test on cells treated with Azacytidine/Decitabine; sick; cells did not undergo normal vegetative division rate, dead; non-viable progenies after refeeding, normal; sexual progenies that underwent normal division rate after refeeding. (c) & (e) IES retention PCRs on different loci (full-length

gels are presented in Supplementary Fig. S3 & S4 respectively) with the primers flanking an IES region (Supplementary Table S1).
