# Supplementary figures and images for: Determination of the presence of 5-methylcytosine in Paramecium tetraurelia
Source: PLoS One. 2018 Oct 31;13(10):e0206667. doi: 10.1371/journal.pone.0206667 (PMC6209305; doi:10.1371/journal.pone.0206667)

S3 Fig

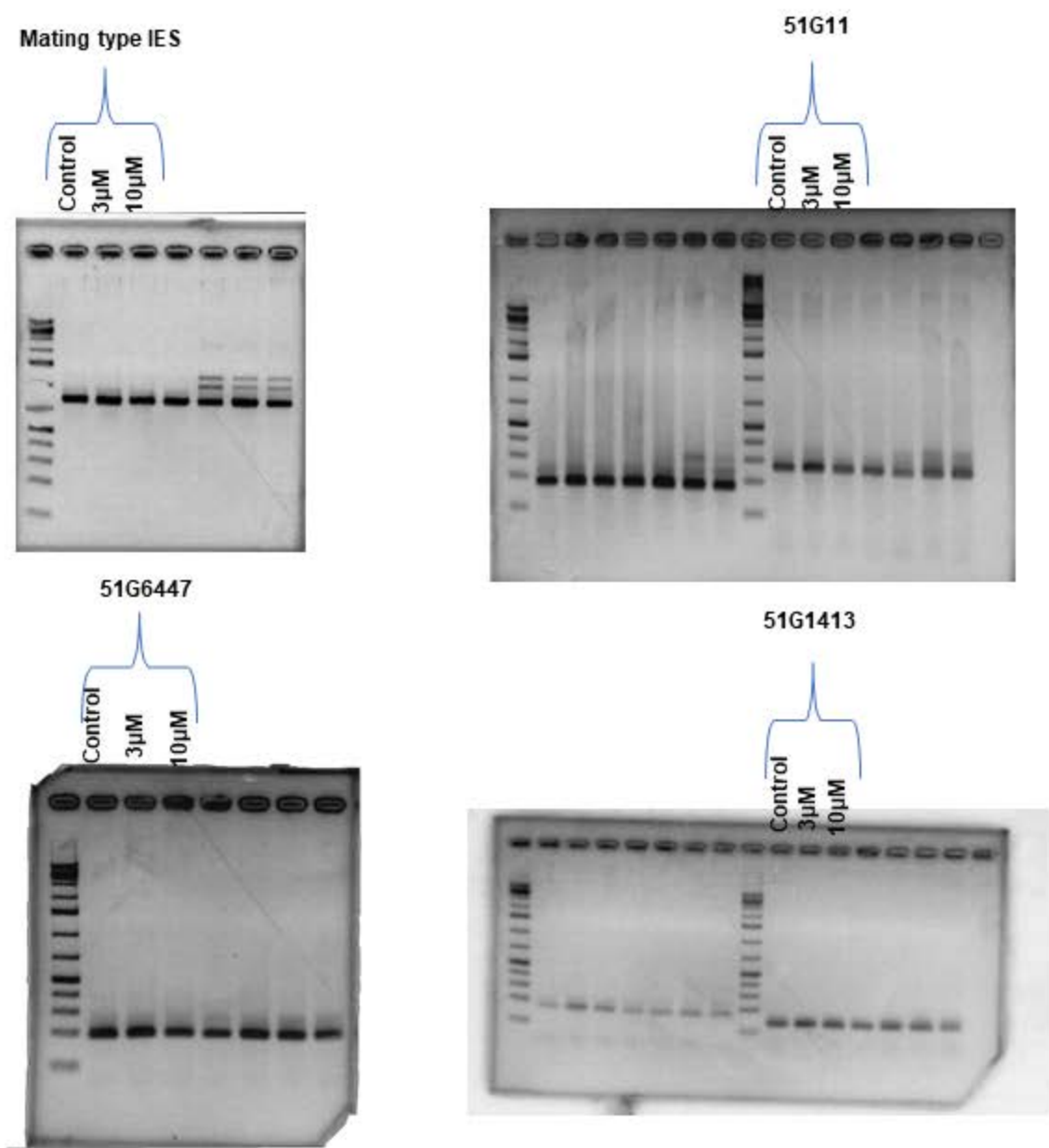

S3 Fig: Full length gels on IES Retention PCRs corresponding to Figure 1c.

Supplement: S3 Fig — (PDF) [file pone.0206667.s003.pdf]

S4 Fig

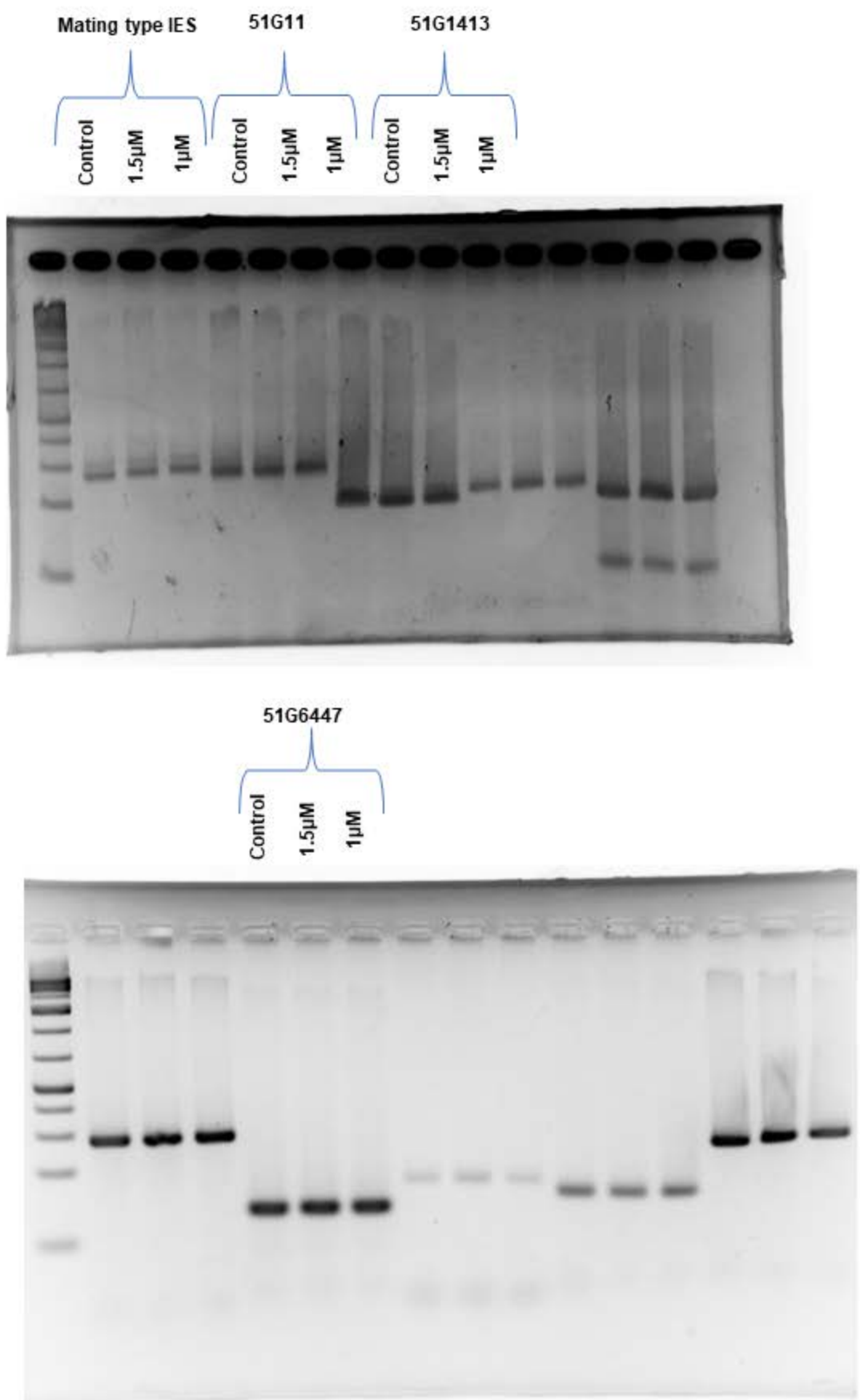

**S4 Fig:** Full length gels on IES Retention PCRs corresponding to Figure 1e.

Supplement: S4 Fig — (PDF) [file pone.0206667.s004.pdf]
