# Supplementary material for: Determination of the presence of 5-methylcytosine in Paramecium tetraurelia
Source: PLoS One. 2018 Oct 31;13(10):e0206667. doi: 10.1371/journal.pone.0206667 (PMC6209305; doi:10.1371/journal.pone.0206667)
Supplement: S5 Fig — Drosophila, E. coli, Human MCF7 DNA and Human T47D DNA (provided by Storm Therapeutics Limited) were used as a positive control for the detection of methylated cytosines. (PDF) [file pone.0206667.s005.pdf]

S5 Fig

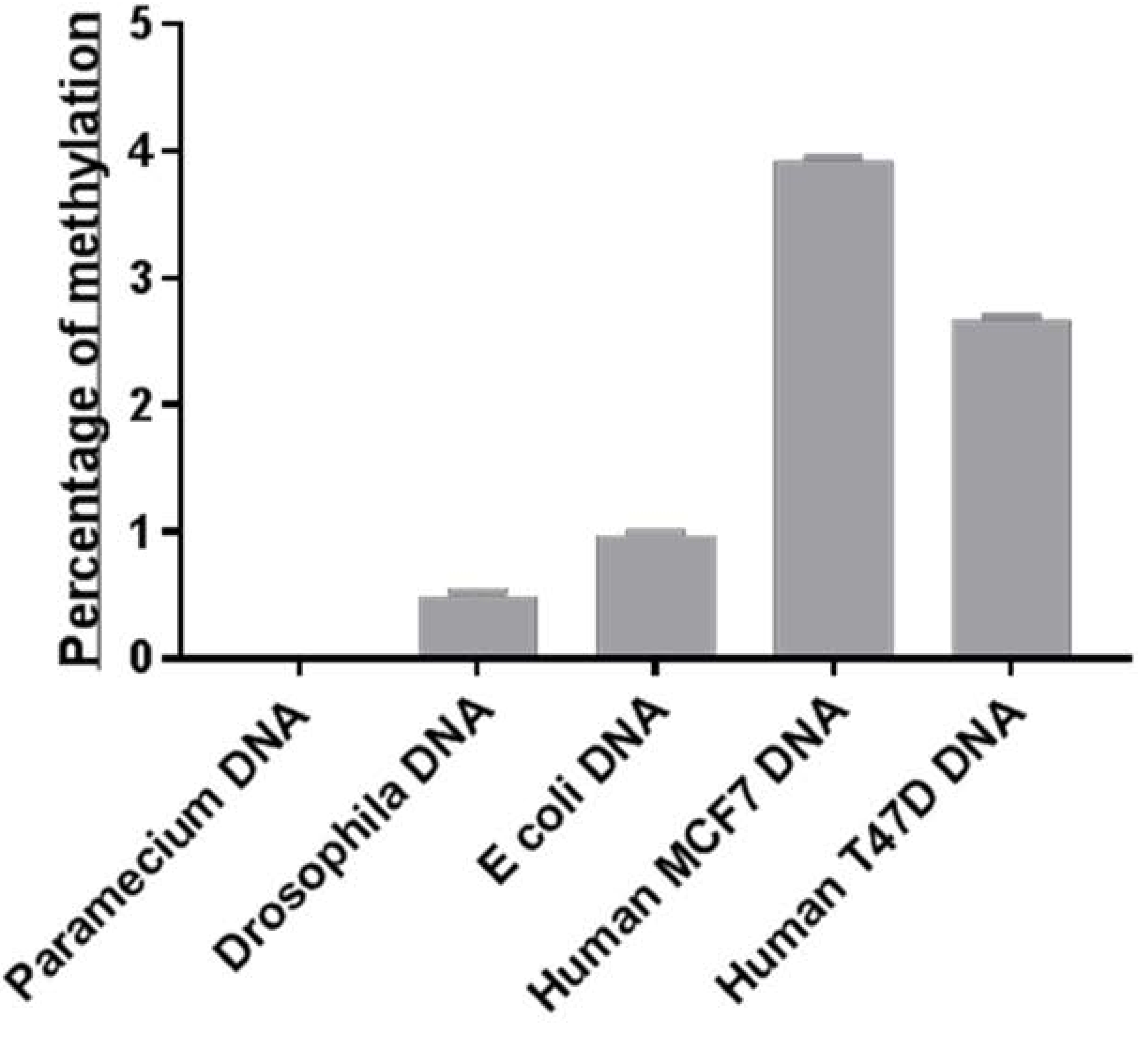

S5 Fig: Percentage of methylation calculated after mass spectrometry done on total genomic DNA samples from different Paramecium during autogamy when new macronucleus are observed in the cell. Drosophila, E. coli, Human MCF7 DNA and Human T47F DNA (provided by Storm Therapeutics Limited) were used as a positive control for the detection of methylated cytosines.
