# Supplementary material for: Determination of the presence of 5-methylcytosine in Paramecium tetraurelia
Source: PLoS One. 2018 Oct 31;13(10):e0206667. doi: 10.1371/journal.pone.0206667 (PMC6209305; doi:10.1371/journal.pone.0206667)
Supplement: S6 Fig — Left panel, percent calculated using C/(C + T) for each C in Paramecium genome. There was no bias in C conversion among the three different nucleotide contexts (right panel). (PDF) [file pone.0206667.s006.pdf]

S6 Fig

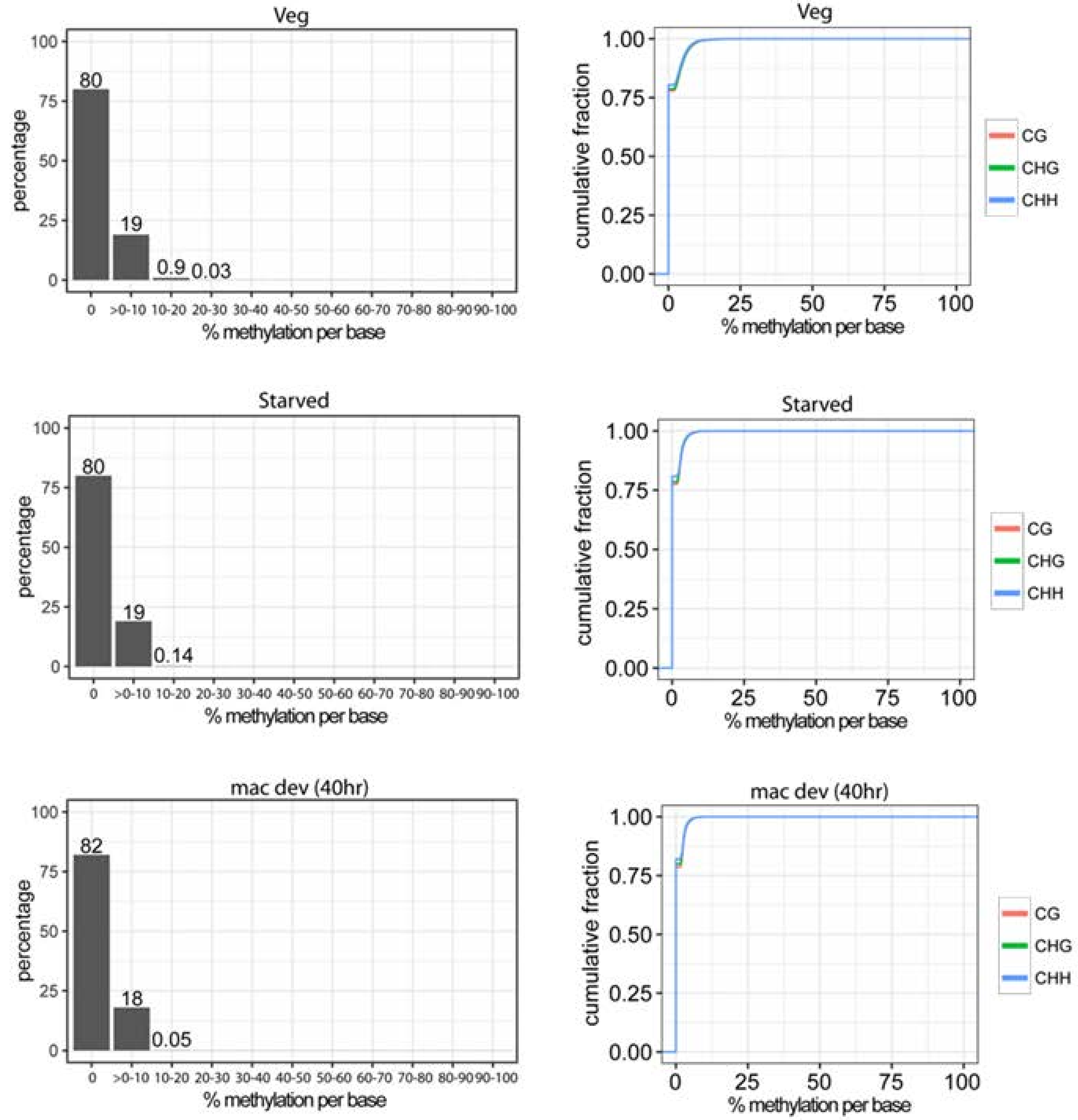

S6 Fig: Absence of evidence of C methylation in *Paramecium* mac genome. Left panel, percent calculated using C/(C + T) for each C in *Paramecium* genome. There was no bias in C conversion among the three different nucleotide contexts (right panel).
