# Supplementary material for: Determination of the presence of 5-methylcytosine in Paramecium tetraurelia
Source: PLoS One. 2018 Oct 31;13(10):e0206667. doi: 10.1371/journal.pone.0206667 (PMC6209305; doi:10.1371/journal.pone.0206667)
Supplement: S1 Table — List of primers to check IES retention PCRs. (PDF) [file pone.0206667.s007.pdf]

S1 Table

IES	Primer sequence (5’ to 3’ orientation)
51G-11F	ATCATAAGATTGATATCTTCTCCCTTCTCC
51G-11R	ACTTGCTACTAAAGCAAGAAACATTGAGAG
51G1413F	GAAGCTGCTTGTGTTAAGAATTCTACTGG
51G1413R	GCATCCAGCACTAGTTGAATTTACTGTAC
51G6447F	AATGCATCAAATGTAGTAACTACTCCTGCT
51G6447R	AATTTGTAAAGTATCCAGCGCAGGCAG
MT Locus F	GGTGTTTATATCTTAATTGTTGACCCTCAC
MT Locus R	CCATCTATACTCCATTCTTTATCTTAATTCAT

S1 Table: List of primers. List of primers to check IES retention PCRs
